# Cancer Survivorship Websites and Resources

**DOI:** 10.6004/jadpro.2012.3.3.5

**Published:** 2012-05-01

**Authors:** Denice Economou, Marcia Grant, Mary McCabe

**Affiliations:** Ms. Economou is Project Director/Senior Research Nurse Specialist at City of Hope, Duarte, California; Dr. Grant is Director of Nursing Research and Education in the Department of Population Sciences at City of Hope, Duarte, California; and Ms. McCabe is Director of the Cancer Survivorship Program at Memorial Sloan-Kettering Cancer Center, New York, New York.

As cancer treatment continues to improve and our strategies to prevent and detect new cancers develop, the number of survivors will continue to grow. As statistics show, these patients will number over 11 million by 2020 (National Cancer Institute [NCI], 2011). As the population of cancer survivors continues to grow, awareness of available resources is essential. Advanced practitioners (APs) are the primary coordinators of follow-up care for these patients. Helping patients and their families navigate the available survivorship resources is an important component of the support APs can provide.

Cancer survivors and their families deal with multiple long-term symptoms and late effects that may affect them throughout the rest of their lives. The Institute of Medicine (IOM) consensus report *From Cancer Patient to Cancer Survivor—Lost in Transition* provides a framework of the essential elements of survivorship care (IOM, 2006). The main components for success include coordination and communication, prevention, surveillance for secondary cancers and recurrence, and management of side effects related to treatment or the disease. Multiple resources are available to provide support to cancer survivors and their families (Table 1).

**Table 1:**
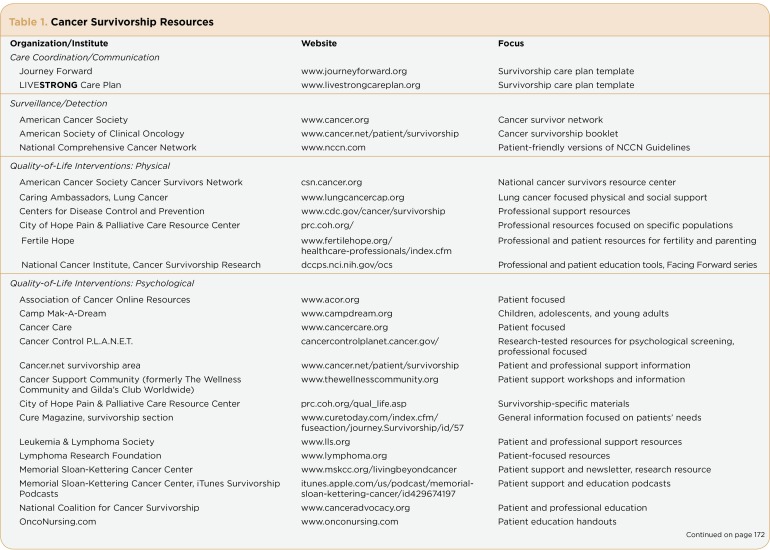
Table 1. Cancer Survivorship Resources

**Table 1:**
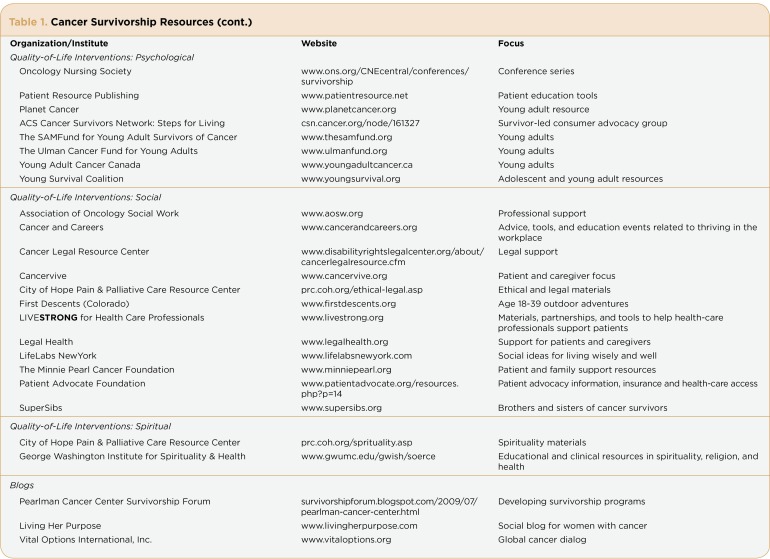
Table 1. Cancer Survivorship Resources (cont'd)

## Coordination and Communication

Resources for coordination and communication in cancer survivorship care include treatment summaries and survivorship care plans. The Commission on Cancer through the American College of Surgeons will require a treatment summary to be provided to all cancer patients by 2015 (American College of Surgeons & Commission on Cancer, 2012).

Some advanced practitioners and physicians are currently providing these plans for patients, but a coordinated multidisciplinary group effort is needed to achieve widespread implementation. As electronic medical records become more prevalent, treatment summaries will be much easier to complete. Unfortunately, electronic medical records are not widespread as of yet, and although it is essential, abstracting the treatment information from the medical record is very time consuming.

There are several websites that can assist patients in creating individualized survivorship care plans. These can be completed by the patient and/or by a professional. Journey Forward (www.journeyforward.com), a program that was developed collaboratively with the National Coalition for Cancer Survivorship, UCLA Cancer Survivorship Center, the Oncology Nursing Society, WellPoint, Inc., and Genentech, is one such resource. Journey Forward’s survivorship care plan builder, which can be accessed through the Internet, helps build a care plan that includes background information, treatment plan and summary, and follow-up care suggestions based on American Society of Clinical Oncology (ASCO) recommendations (Journey Forward, 2012). The LIVESTRONG Care Plan is another website-based care plan builder that has been updated using the OncoLink format with the University of Pennsylvania. Both of these resources offer patient educational materials as well as care plan follow-up recommendations.

A recognized key to success in care planning is beginning at diagnosis. These tools can be started as treatment begins and built upon throughout the treatment course. Encourage patients to keep a diary of treatments, and help your setting provide proactive treatment summaries to patients as they begin treatment. These steps will improve health-care providers’ ability to coordinate follow-up care.

## Surveillance, Detection, and Risk Reduction

Resources related to surveillance for new cancers and detection of recurrence are primarily based on the American Cancer Society guidelines for cancer screening. Surveillance protocols after a cancer diagnosis vary among some tumor sites with known recurrence risks, such as breast and prostate cancers. No standardized guidelines exist at this time, so follow-up surveillance is based on Amercian Cancer Society, ASCO, and National Comprehensive Cancer Network (NCCN) recommendations for detection, prevention, and risk reduction. The NCCN provides guidelines in these areas for certain cancers, including breast, cervical, colorectal, lung, and prostate, as well as for genetic/familial high-risk assessment for breast and ovarian cancers (NCCN, 2012). Individual physicians may direct their patients’ follow-up surveillance as desired.

## Interventions

Resources for interventions are organized around the quality-of-life model for a cancer survivor, which includes physical, psychological, social, and spiritual domains. The City of Hope Pain & Palliative Care Resource Center (PRC) provides many resource publications, tools, and websites for cancer survivorship as well as pain, palliative care, and spiritual resources (City of Hope PRC, 2012). The NCI website http://dccps.nci.nih.gov/ocs/ includes research resources as well as educational tools. Psychological and social resources offer access to professional as well as peer support.

Finally, there are growing numbers of blogs and other Internet resources for patient and caregiver support. The Pearlman Cancer Center blog was developed to help connect survivorship programs and share program-building resources and ideas. Many cancer survivors are very Internet-literate and use the Internet to connect to their peers. Memorial Sloan-Kettering Cancer Center’s website includes a newsletter written by its survivors. It is an example of how survivors with professional experience can use their talents to reach out to other survivors.

## Conclusions

The growing survivor population will continue to require AP support as the general population ages. Cancer survivors with multiple comorbidities need care and guidance. Integrating survivorship care from diagnosis through treatment and follow-up can lead to quality cancer care for patients and their families.
